# Accurate Phylogenetic Relationships Among *Mycobacterium bovis* Strains Circulating in France Based on Whole Genome Sequencing and Single Nucleotide Polymorphism Analysis

**DOI:** 10.3389/fmicb.2019.00955

**Published:** 2019-05-03

**Authors:** Amandine Hauer, Lorraine Michelet, Thierry Cochard, Maxime Branger, Javier Nunez, Maria-Laura Boschiroli, Franck Biet

**Affiliations:** ^1^University Paris-Est – ANSES, French Reference Laboratory for Tuberculosis, Maisons-Alfort, France; ^2^ISP, INRA, UMR 1282, Université de Tours, Nouzilly, France; ^3^Animal and Plant Health Agency, Addlestone, United Kingdom

**Keywords:** *Mycobacterium bovis*, bovine tuberculosis, phylogenetic, SNP, WGS, evolution

## Abstract

In recent years the diversity of the French *Mycobacterium bovis* population responsible for bovine tuberculosis (bTB) outbreaks since 1970 has been described in detail. To further understand bTB evolution in France, we used single nucleotide polymorphisms (SNPs) based on whole genome sequence versus classical genotyping methods in order to identify accurate phylogenetic relationships between *M. bovis* strains. Whole genome sequencing was carried out on a selection of 87 strains which reflect the French *M. bovis* population’s genetic diversity. Sequences were compared to the *M. bovis* reference genome AF2122/97. Comparison among the 87 genomes revealed 9,170 sites where at least one strain shows a SNP with respect to the reference genome; 1,172 are intergenic and 7,998 in coding sequences, of which 2,880 are synonymous and 5,118 non-synonymous. SNP-based phylogenetic analysis using these 9,170 SNP is congruent with the cluster defined by spoligotyping and multilocus variable number of tandem repeat analysis typing. In addition, some SNPs were identified as specific to genotypic groups. These findings suggest new SNP targets that can be used for the development of high-resolving methods for genotyping as well as for studying *M. bovis* evolution and transmission patterns. The detection of non-synonymous SNPs on virulence genes enabled us to distinguish different clusters. Our results seem to indicate that genetically differentiated clusters could also display distinctive phenotypic traits.

## Introduction

*Mycobacterium bovis*, the main etiological agent of bovine tuberculosis (bTB), is a member of the *Mycobacterium tuberculosis* complex (MTBC) (Huard et al., 2006; Smith et al., 2006a,b). This highly clonal species, which derived from a common ancestor related to *Mycobacterium canettii* (Blouin et al., 2012), includes seven different members: *M. tuberculosis*, *M. bovis*,*M. africanum*, *M. microti*, *M. caprae*, *M. canettii*, *M. pinnipedii*. Members of the *M. tuberculosis* complex may be considered a series of host-adapted ecotypes rather than species that are part of a strain lineage (Smith et al., 2006a, 2009). These ecotypes are characterized by the absence of a chromosomal region. *M. bovis* is the final ecotype of a series of evolution from an ancestor related to *M. tuberculosis*. To date, four clonal complexes of *M. bovis* have been defined on the basis of deletions, distinct spoligotype and SNPs signatures: European 1, European 2, African 1, African 2 (Muller et al., 2009; Smith, 2012; Rodriguez-Campos et al., 2014). In France, as in many countries, bTB has a great economic impact due to the high cost of the control and eradication campaign as well as the impossibility to trade live animals or animal products (Anonymous, 2000; Amanfu, 2006). Furthermore, *M. bovis* infection is a zoonosis and a major concern at the animal-human interface, mainly through the consumption of raw milk or raw milk products (Palmer et al., 2012; Muller et al., 2013) particularly in those regions of the world where predisposing factors such as poverty or aids prevail.

Genetic differentiation of strains has become an indispensable tool to study the evolution, epidemiology and ecology of this pathogen.

For epidemiological surveillance, spoligotyping and the multilocus variable number of tandem repeat analysis (MLVA), two molecular tools that make it possible to discriminate strains by their genotypes (Aranaz et al., 1996; Skuce et al., 2005; Allix et al., 2006; Supply et al., 2006) are currently used worldwide (Haddad et al., 2001; Boniotti et al., 2009; Rodriguez et al., 2010; Smith, 2012; Rodriguez-Campos et al., 2013). Using these conventional methods, we recently described the dynamic genetic evolution of the French *M. bovis* population studying strains isolated mainly from livestock and wildlife that has been isolated since 1978 (Hauer et al., 2015). During this period, the number of bTB-positive herds decreased from 15% to less than 0.1% although outbreaks persisted in some regions. This decrease in prevalence was concomitant with a decrease of the genetic diversity of strains characterized by a strong regionalization and the evidence of multi-host transmission cycles in which both livestock and wildlife are infected by strains of the same genotype. The observed genetic relationships between the strains supports the co-existence of at least four clonal populations within the French *M. bovis* population (Hauer et al., 2015).

Whilst spoligotyping and MLVA are unquestioned tools for disease outbreak investigations, recent studies show the limitations of these methods for tracing the origin of a given infection (Rodriguez-Campos et al., 2011). Although these typing techniques target polymorphic genetic regions, they interrogate less than 1% of the genome and therefore have an intrinsically restricted discriminatory power. Furthermore regarding spoligotyping, the deletion of the same type of spacer can occur in entirely separate sub-lineages (Corner et al., 1995). Likewise, copy numbers of MIRU (Mycobacterial Interspersed Repetitive Units) – VNTR (Variable Numbers of Tandem Repeats) at each locus can increase or decrease during DNA replication which may contribute to give rise to identical patterns in separate sub-lineages (Corner et al., 1995).

These limitations can be overcome by the application of whole genome sequencing (WGS) for genome-based epidemiology especially for clonal populations belonging to highly concordant phylogenies with low homoplasy. WGS can provide comprehensive genetic information including all possible genomic targets as well as additional information on genome evolution, virulent determinants and drug resistance traits. Using the WGS, single nucleotide polymorphisms (SNPs) analyses become a robust tool for studying closely related strains of pathogenic mycobacteria (Garcia Pelayo et al., 2009; Homolka et al., 2012; Joshi et al., 2012; Roetzer et al., 2013).

The purpose of the present study was to undertake SNP analysis based on WGS versus classic genotyping for identifying accurate phylogenetic relationships within the population of French *M. bovis* strains. A set of 87 animal field strains representative of the French *M. bovis* population’s genetic diversity was selected based on a previous large-scale genotyping study (Hauer et al., 2015). We performed WGS of the 87 field isolates to detect SNPs by genome comparison among these *M. bovis* strains using the genome *M. bovis* AF2122/97 as reference. The SNP analysis was addressed to verify the accuracy of the phylogenetic lineages of the isolates and to establish correlations between genotypes and phenotypes based on particular SNP profiles targeting virulence genes.

## Materials and Methods

### *Mycobacterium bovis* Strains

A panel of 87 *M. bovis* isolates from French bovine tuberculosis outbreaks occurring between 1978 and 2011 (Table 1 and Supplementary Table S1) was carefully selected so as to represent the genetic diversity of the main genotypes described in our recent previous study. Isolates were obtained from animals studied in the framework of French bTB control campaign from different hosts: wild boars (*n* = 2); pig (*n* = 1); sheep (*n* = 1); goat (*n* = 1); deer (*n* = 1) and mostly from cattle (*n* = 81). Details of the spoligotype and MLVA profiles of the isolates selected to be sequenced are indicated in Table 1 and Supplementary Figure S1. The following six query genomes were also included as references: *M. bovis* AF2122/97_BX24833 (EMBL sequence AF2122/97_BX248333); *M. tuberculosis* H37Rv (NC_000962.2); *M. africanum* MAL010084 (JKXR00000000.1); *M. canettii* CIPT 140010059 (NC_015848.1); *M. bovis* BCG Pasteur_1173P2 (NC_008769.1); and *M. bovis* BCG Tokyo_172 (NC_012207.1).

**Table 1:** Details on strains used in this study.

Genetic group and strain Field strains	No. of strains sequenced	International spoligotype ID	MLVA profiles ID	B	Wb	D	C	P	O
SB0120	28	SB0120	23	22	1	1			
		SB0822	1	1					
		SB0828	2	2					
		SB0948	1	1					
SB0121 (Eu2 clonal complex)	17	SB0121	11	10					1
		SB0119	1	1					
		SB0295	1	1					
		SB0837	1	1					
		SB0839	2	2					
		SB0999	1	1					
F4	11	SB0818	1	1					
		SB0821	2	2					
		SB0823	1	1					
		SB0825	1	1					
		SB0826	1	1					
		SB0827	1	1					
		SB0840	1					1	
		SB0843	1	1					
		SB0928	1	1					
		SB0946	1	1					
F9	6	SB0819	4	4					
		SB0830	1	1					
		SB0853	1	1					
SB0134	11	SB0134	7	6	1				
		SB0089	2	2					
		SB0128	1	1					
		SB0824	1	1					
Eu1 clonal complex	6	SB0140	4	4					
		SB0130	1	1					
		SB0145	1	1					
Others	8	(*n* = 7) see TS1	8	7			1		
Sub total	87^a^	37	86	81	2	1	1	1	1
**Reference strains**^b^	7								
*M. bovis* AF2122	1	SB0140	1						
*M. bovis* AN5	1	SB0268	1						
BCG Pasteur	1	SB0120	1						
BCG Tokyo	1	SB0120	1						
*M. africanum* MAL010084	1	SIT181	1						
*M. canettii* CIPT 140010059	1	SIT592	1						
*M. tuberculosis* H37Rv	1	SIT451	1						
Total	94								

*^a^Sequenced in this study, ^b^previously sequenced. B, bovine; Wb, wild boar; D, deer; C, caprine; P, pig; O, ovine; SIT, shared international type.*

### DNA Extraction

Clones of the different isolates were enriched in 50 mL Middlebrook 7H9 broth and harvested at mid log phase growth and pelleted at room temperature at 5,500 g. Pellets were inactivated by heating at 80°C during 30 min. DNA was extracted by a chloroform protocol with a lysozyme pre-step followed by a proteinase K treatment. CTAB/NACL was added before the chloroform/isoamyl alcohol action. Precipitation of DNA was performed in isopropanol. DNA was conserved at -20°C in 1x TE buffer (Imai et al., 1994; Biek et al., 2012).

### Genotyping

Spoligotyping of 43 spacers sequences contained in the direct repeat (DR) locus was performed on a Luminex (Zhang et al., 2010). Spoligotypes were named according to an agreed international convention^1^ (Smith, 2012). MLVA typing (Frothingham and Meeker-O’Connell, 1998; Roring et al., 2002) was performed by Genoscreen (Lille, France), on 8 MIRU – VNTR loci: ETR A (2165), ETR B (2461), ETR C (577), ETR D (580), QUB 11a (2163a), QUB 11b (2163b), QUB 26 (4052), QUB 3232 (3232) chosen upon the Venomyc European consortium and described by Hauer et al. (2015, 2016).

### Whole Genome Sequencing of French *M. bovis* Field Isolates

Whole genome paired end (2 × 100 bp) sequencing was performed using Illumina HiSeq technology by Genoscreen (Lille, France) (Supplementary Table S2). To avoid PCR overrepresented fragments during the library preparation, the paired-end FASTQ files were filtered from replicated sequences, leaving only one pair of short reads for each set of pair of replicated short reads.

The short reads were trimmed using Trimmomatic (Bolger et al., 2014) when the quality call given by the sequencer (range: 0 to 40) across the sequence dropped lower than 20 for a slidding window of 10bp (Bolger et al., 2014). The minimum read length threshold equal 35 bases. The resulting mean length of the short reads per sample ranged from 95.31 to 98.42 bp with standard deviation ranging from 10.25 to 13.78 (Supplementary Table S2).

The FASTQ were then mapped against the *M. bovis* reference strain AF2122/97-BX24833 using SMALT (Sanger Institute). The mean coverage or depth obtained ranged from 27 to 105 and the similarity base to base to the genome reference ranged from 98.5 to 99.6% (Supplementary Table S2). Both parameters indicate an acceptable quality to perform variant calling over the full genome.

*Mycobacterium bovis* BCG Pasteur_1173P2 (NC_008769.1) and *M. bovis* BCG Tokyo_172 (NC_012207.1) were included with the 87 genomes for SNP analysis.

Data deposition: the 87 genomes have been deposited at GenBank under the Accession Nos. MINA00000000.1, MTZQ00000000, MTZS00000000, MTZR00000000, and under submission SUB4397761 to be released upon publication (Bioproject Accession No. PRJNA485121) see also Supplementary Table S1.

### SNP Identification and Selection

The comparison of the 89 genomic sequences led to the identification of SNP mutations. SNP were screened using information in the whole genome including intergenic, synonymous, and non-synonymous mutations.

SAMtools and BCFtools were used to transform the resulting BAM files format into VCF files. Then three tests were used to identify reliable SNPs; SNPs have a minimum of SAMtools quality score of 150. The number of forward (reverse) reads mapping with good quality onto the SNP site having the same base as in the reference had to be less than 20% of the total number of forward (reverse) reads mapping with good quality onto the SNP site having the variant base and the minimum forward (reverse) coverage on a SNP site has to be greater than 5. A total of 123 SNPs found in repetitive regions were filtered out. To perform this task, a window of 50 bp around the SNPs sites were blasted against the reference genome. The SNPs corresponding to sequences with more than one blast-hit (>99% match) were filtered out.

Single nucleotide polymorphisms distant on less than 10 bp from one another were excluded as they may be due to sequencing mistakes, as described before (Gardy et al., 2011).

### Phylogenetic Analysis

Multilocus variable number of tandem repeat analysis and spoligotype patterns were carried out using Bionumerics 7.6.2 software (Applied Maths, Sint-Martens-Latem, Belgium) by drawing minimum spanning trees (MSTs) and dendrograms [Categorical (values) distance represented by UPGMA clustering method for MLVA or spoligotype, Multi-state categorical correlation represented by UPGMA clustering method for MLVA plus spoligotype]. SNP data were concatenated, resulting in a single character string (nucleotide sequence) for each strain. Phylogenetic and molecular evolutionary analyses were conducted using MEGA (version 7^2^), using the neighbor-joining method or maximum likelihood method with 1,000 bootstrap replicates, with distance calculated using the number of different SNPs.

### SNP on Virulence Genes

We investigated whether it was possible to identify virulence traits related to the different phylogenetic lineages of French *M. bovis* by SNP analysis. To do this, we searched for SNP mutations among 181 genes that were described to be essential for virulence of the MTBC. The list of genes included in this study (Supplementary Table S4) were selected from the studies of Sassetti and Rubin (2003); Joshi et al. (2006), and Forrellad et al. (2013). Based on their function, molecular features or cellular localization, these 181 target genes included the following categories (Supplementary Table S4): Lipid and fatty acid metabolism (*n* = 25), Catabolism of cholesterol (*n* = 8), Cell envelope proteins (*n* = 12), ‘mce’ families proteins (*n* = 21), Lipoproproteins (*n* = 6), Metal-transporter proteins (*n* = 9), PE and PE_PGRS families regulators (*n* = 12), Secretion system (*n* = 17), Proteases (*n* = 6), Genes and metabolisms regulation expression (*n* = 26), Defense mechanisms (*n* = 30), and Other unknown function (*n* = 9).

### Homoplasy Index Calculation

The homoplasy index was calculated with software package PAUP^∗^ (Phylogenetic Analysis Using Parsimony version 4) (Wilgenbusch and Swofford, 2003)^3^.

## Results

### Global *MTBC* Phylogeny Picture

**FIGURE 1:**
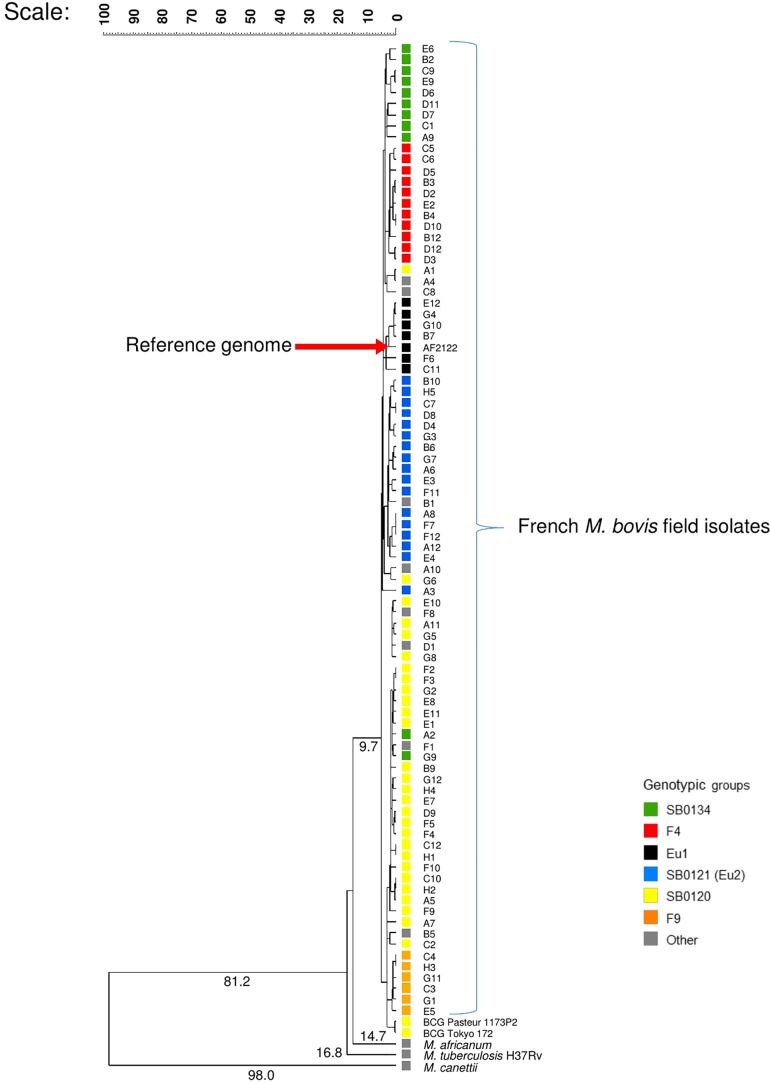
Whole genome-based evolutionary analysis of the panel of French field isolates of *M*ycobacterium *bovis*. The phylogenetic tree with numbered above the branches indicating the similarity coefficient between the organism and its common ancestor was constructed with whole genome, using the software bionumerics vs. 7.6.2 with the settings: similarity coefficient by categorical, scaling factor of 100 and clustering method by UPGMA.

Whole genome sequencing was obtained for 87 *M. bovis* isolates covering the genetic diversity of French strains previously distributed in six groups (SB0120 = 28; Eu2 = 17; SB0134 = 11; F4-family = 11; F9-family = 6; Eu1 = 6; Others = 8) (Hauer et al., 2015). In a first analysis, members of MTBC were included to place the 87 French *M. bovis* field isolates in a wider mycobacterial context as shown in the Figure 1.

The sequence reads of each strain were mapped to the assembled genome of *M. bovis* AF2122/97 to determine a similarity coefficient based on categorical difference. The clustering method UPGMA was used to depict the evolutionary relationships between the French *M. bovis* field isolates’ genomes and the other genomes of the MTBC members presented in Figure 1.

Consistent with the predicted evolutionary scenario, the phylogenetic analysis showed *M. canettii*, is the most distantly related to the *M. bovis* strains with a coefficient of 98 followed by *M. tuberculosis* and *M. africanum*, the two preferentially human strains. *M. bovis* field isolates are closely related, nonetheless well-differentiated groups were distinguished. The BCG strains and those of the SB0120 group that share the same spoligotype appeared to be closely related and more distant to the strain AF2122/97 used as reference.

### WGS and SNP Analysis

Whole-genome sequences were analyzed in order to detect SNP in comparison with the reference genome of *M. bovis* AF2122/97 allowing us to identify possible phylogenetic lineage markers and thus to resolve the phylogeny within *M. bovis* field isolates. The new 87 genomes sequences of the *M. bovis* field isolates plus the sequences of reference strains (*M. bovis* Pasteur_1173P2 BCG; *M. bovis* Tokyo_172 BCG) were mapped to the *M. bovis* reference strain (AF2122/97-BX24833) to identify SNPs.

**FIGURE 2:**
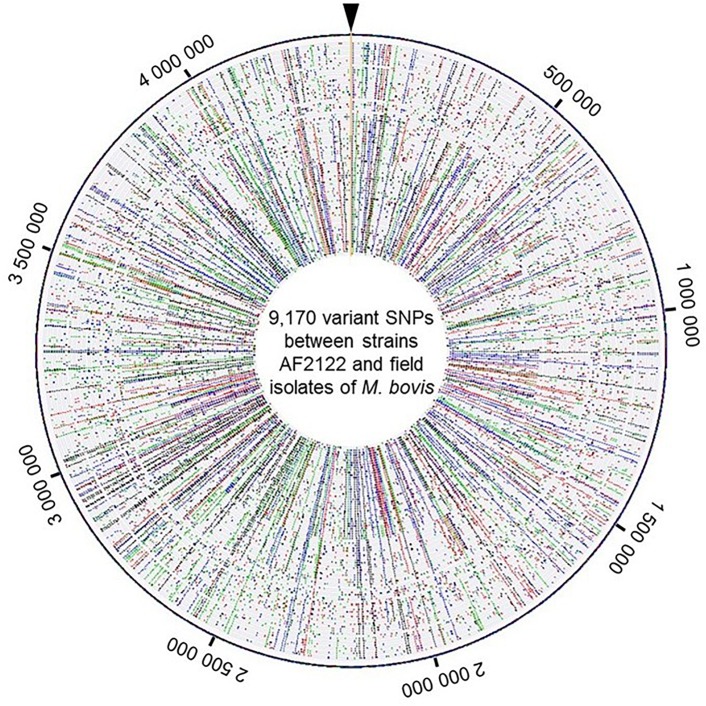
View of the genome wide distribution of the 9,170 variant SNPs identified between the strains AF2122/97 and the 87 field isolates of *M. bovis*. Maps were generated using Bionumerics software. Each circle corresponds to one strain genome. The field isolate genomes were arranged by genotype group from the reference genome depicted at the outer circle. Around the outer circle are indicated the genome positions (base pair). The base changes are shown in different colors: A in green, C in blue, G in black, and T in red.

A total of 9,170 SNPs were identified, 87% were located in coding regions, 2,880 of them were silent and 5,118 were missense. The distribution of SNPs covers the whole chromosome as depicted in Figure 2 and detailed in Supplementary Table S3.

In previous works, six groups, including the Eu 1 and Eu 2 clonal complexes, were distinguished within the population of *M. bovis* strains present in France (Rodriguez-Campos et al., 2012, 2014; Smith, 2012; Hauer et al., 2015). These groups were defined by the combination of their spoligotypes, MLVA patterns and other mutations (summarized in Table 2).

**Table 2:** Definition and characteristics of the phylogenetic groups of *M. bovis* identified in France.

Groups	Denomination	Strain number	frequency in France	Spoligotype marker: spacer absent	MLVA marker	WGS	Specific SNPs in this study
A	F4	11	25.6%	33	QUB26 truncated, QUB3232 more variable		74
B	ND	3	ND	ND	ND		52
C	SB0134	9	14.2%	4–5	QUB26, QUB11a more variables		7
D	Eu1	6	1%	11	ND	RDEu1	58
E	ND	3	ND	ND	ND		6
F	Eu2	17	7%	21	QUB3232, QUB11a more variables	guaA mutation	95
G	F9	6	0.4%	1–17 and 23–24	ND		98
H	ND	2	ND	ND	ND		58
I	SB0120	30	49.4%		ETR-B, QUB11a more variables		5

*ND, not documented.*

*From Hauer et al. (2015).*

**FIGURE 3:**
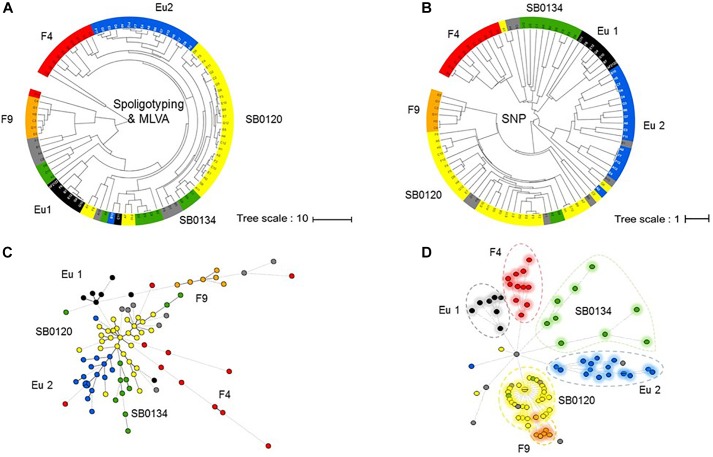
Phylogeny of the French *M. bovis* field isolates resolved with SNPs. UPGMA phylogenetic tree based on a combination of spoligotyping and MLVA **(A)** or inferred from the alignment of the pseudosequences of the 9,170 SNPs in the 87 genome **(B)**. Minimum spanning tree (MST) based on combination of spoligotyping and MLVA **(C)** and genome-wide SNP **(D)** of the 87 French isolates plus the genome of the reference strain AF2122/97. The genotype groups defined in Table 1 are represented by name and their associated color.

The established phylogenetic analysis based on SNPs categorized all *M. bovis* isolates into groups which are congruent with those previously established with the combination of spoligotyping and MLVA (Figure 3A,B).

The results presented by the MST in Figure 3C,D show that the group resolution is better with SNPs analyses in comparison to combination of spoligotyping and MLVA. These observations suggest that SNP are better phylogenetic markers for *M. bovis*.

Not surprisingly, we observe that the highest homoplasy rate is given by spoligotyping (0.647) followed by MLVA (0.578) and significantly lower (0.004) by the use of SNPs (Supplementary Figure S2).

In addition, these analysis show that all the strains which clustered in the European 2 complex possessed the mutation in *gua*A (G→A; genome position 3765573 and gene position 843) unique of this complex (Rodriguez-Campos et al., 2012). From the set of SNPs listed in Garcia Pelayo et al. (2009), 300 were identified, in at least one strains, in our study. The details of their position and the genes impacted are indicated in the Supplementary Table S8.

### Phylogenetically Informative SNPs

**FIGURE 4:**
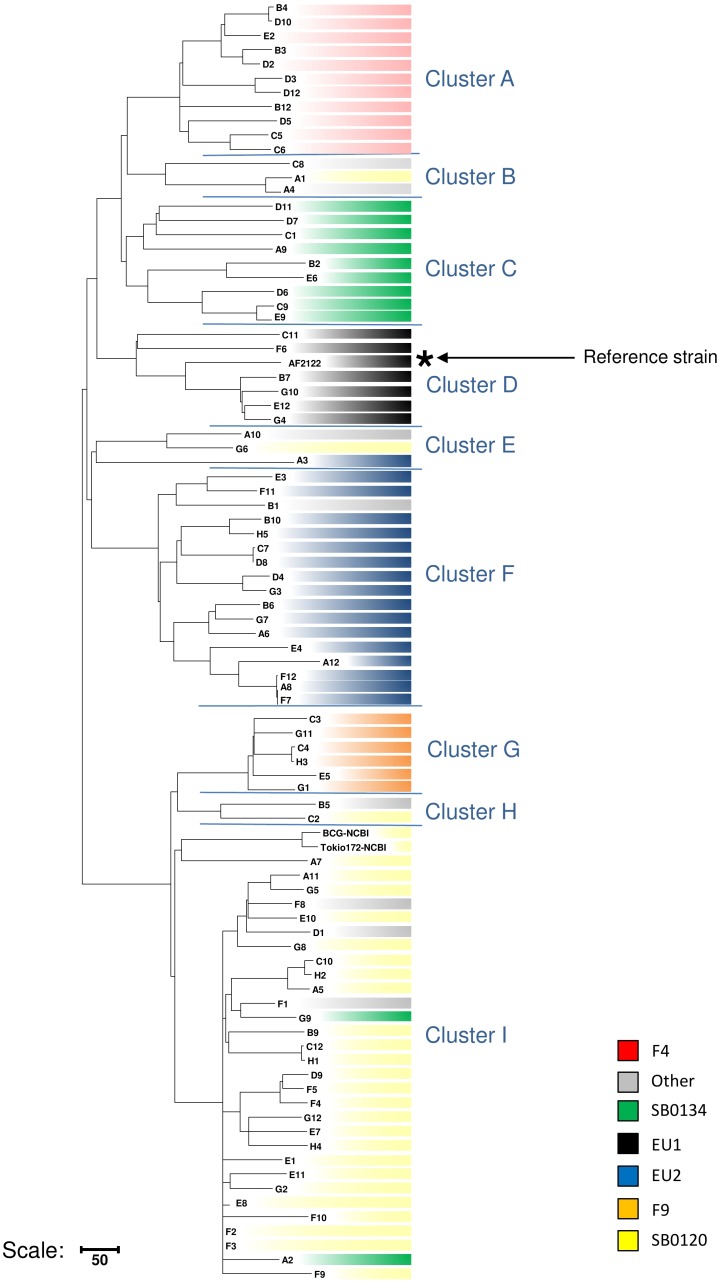
Detail of number of SNPs discriminating the cluster of strains. Maximum parsimony tree based on the 9,170 SNPs identified through the mapping of 87 French *M. bovis* sequences on AF2122/97 reference sequence created using MEGA4 (Tamura et al., 2007). Numbers indicated on the branches represent the numbers of SNP. The tree is subdivided in nine main clusters described in the text.

The dendrogram presented in Figure 4 details the numbers of SNP separating each sequenced strain distributed in the nine clusters, six of which have been previously described in the literature (Rodriguez-Campos et al., 2012; Smith, 2012; Hauer et al., 2015) and three less representative new groups. On a total of 9,170 SNPs, we established a catalog of SNPs specific to each clonal family (Figure 4 and Supplementary Table S3). Cluster A (F4 group), previously defined by the lack of spacer 33 in the DR region and by a truncated repetition of the QUB26 locus, possesses 74 specific SNPs and is closely related to a new cluster B, defined by 52 specific SNPs, which is composed by only three strains from our selection. The next group on the phylogenetic tree is C (SB0134 group), which was previously characterized by the absence of the spacer 4 and 5 in the DR region, is only defined by seven SNPs but seems to be divided in two well-defined (respectively 28 and 29 specific SNPs) and balanced (respectively 4 and 5 strains) sub-groups. The last cluster on this branch includes the Eu1 complex, previously characterized by the absence of the spacer 11 and the RDEu1 deletion, which is defined here by 58 specific SNPs and composed by six of our strains and the AF2122/97 reference sequence. Another branch of the phylogenetic tree is composed by two cluster: a new one, “E,” only composed by three strains and defined by only six specific SNPs, and the well-defined (95 specific SNPs) Eu2 complex, also characterized by the absence of the spacer 21 in the DR locus and the *guaA* mutation (genome position 3765573 and gene position 843). The last and well-separated branch (119 SNPs) is subdivided into three different clusters. Cluster G (F9 group), defined by 98 SNPs, is closely related to a new cluster, cluster H, which is composed by only two strains from our selection. Cluster I (SB0120 group) is only separated from the two previous ones by five SNPs. This cluster is composed by 30 field strains and two BCG reference sequences. The reference sequences clustered with one field strain (A7) and are well-separated from the other field strains, which form a clear group defined by 66 SNPs. This analysis confirms the coexistence of several groups in France, some of them already defined as clonal groups. Three of them represent almost 90% of the French strains: group I (SB0120), the largest one with 44.7% of the strains, and group A (F4 family) and group C (SB0134 group), with 28.5% and 14.5% respectively. Three other groups had been described before but are less preponderant in the French population: the Eu2 complex, dominant in the Iberian Peninsula, represents 9% of the French strains; the Eu1 complex, dominant in the British Islands, represents 1% of the French diversity; finally the group G (F9 family), a small group representing only 0.3% of the French diversity but clearly defined by a particular spoligotype pattern (absence of spacer 1–17 and 23–34) and localized in the south west of France. The new groups B, E, and H are not defined by any other characteristics on their spoligotype pattern or MLVA markers but regarding the phylogenetic analysis they seem to be clearly separated from the other groups. Further analysis on a larger set of strains is necessary to better define these groups.

These results suggest that SNPs that are able to distinguish each cluster can be used to develop a novel SNP-based MLST to facilitate global epidemiology studies.

### SNP: From Phylogeny to Virulence Traits

**FIGURE 5:**
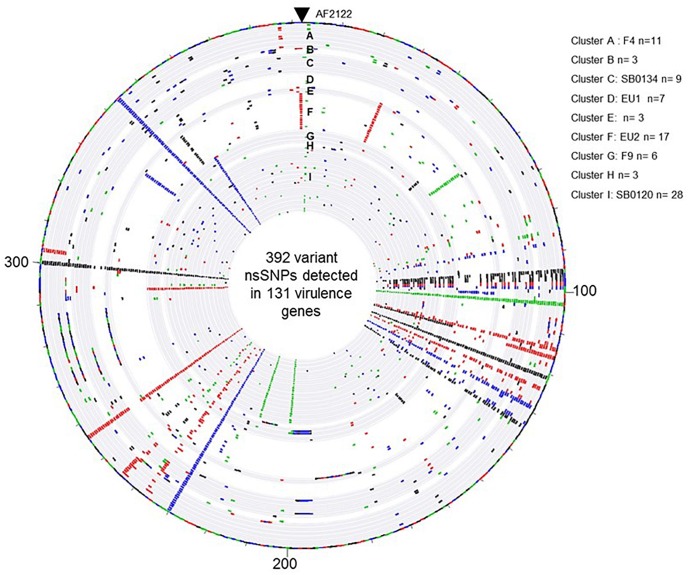
Overview of the distribution of the 392 variant non-synonymous SNPs detected in the 131 virulence genes by comparison of the *M. bovis* strain AF2122/97 and the 87 French *M. bovis* field isolates. The map was generated with Bionumerics software. Each circle corresponds to a pseudosequence of the polymorphic 392 non-synonymous SNPs detected on the 131 virulence genes of one strain. From outer to inner: strain AF2122/97 then the 87 field isolates arranged by their genetic Cluster, from A to I. The base changes are shown, A in green, C in blue, G in black, and T in red.

**FIGURE 6:**
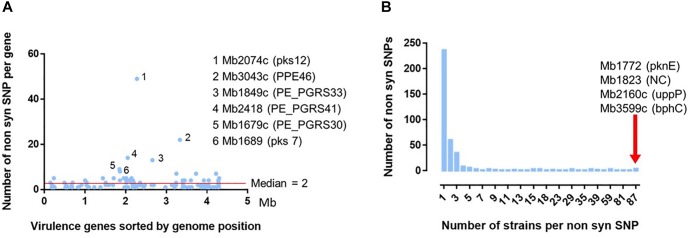
Analysis of the 392 non-synonymous SNP detected in 131 virulence genes on the 87 French *M. bovis* isolates. **(A)** Distribution of the 392 non-synomymous SNPs detected in the French isolates in comparison to the reference genome of *M. bovis* AF2122/97. The position of the detected nsSNPs was ordered according the genomic location of the 131 virulence genes (detailed in Supplementary Table S4). The six genes with the non-synonymous SNP most frequently detected are indicated with their locus tag and their acronym (in brackets). **(B)** Distribution of number of strains per non-synonymous SNP detected on 131 virulence genes. Most of the 392 ns SNPs are detected on one unique strain. The red arrow indicates four non-synonymous SNPs, in four different genes indicated by their locus name and acronym, present in the totality of the 87 French field isolates compared to the reference genome AF2122/97.

In order to learn if the above described groups could present differential virulence traits, the non-synonymous SNP (nsSNP) variants were investigated on 181 virulence genes (Supplementary Table S5). On these 181 genes, 30 were not affected in any strain genome (Supplementary Table S6), 151 genes have at least one SNP and in total 568 SNP, 176 synonymous and 392 non-synonymous, were identified (Supplementary Table S5). An overview of the distribution of the non-synonymous SNP (nsSNP) detected in each strain, organized by group, is illustrated in Figure 5. Three situations could be observed. In the first one, SNPs are distributed randomly (most frequent case see Figure 5). In the second situation, the same mutations are found in all French isolates at the same position (*n* = 4) (four full rays in the Figure 5, 6B) indicating that these four SNP are specific of the AF2122/97 reference strain. The third case, which is the most interesting one, shows mutations that are specific to either several or only just one family (partial concentric line). As shown in Figure 6B the vast majority of nsSNPs were detected in only one genome (236/392).

Some genes represent a higher degree of mutation (rate median = 2) (Figure 6A). This is particularly the case of six genes (Supplementary Table S7): Mb2074c (pKS12, 12457 amino acid) with 49 SNP variants, Mb3043c (PPE46, 1315 amino acids) 22 variants, Mb1849c (PE-PGRS33, 1507 amino acids) 14 variants, Mb2418 (PE_PGRS41, 1084 amino acids) 13 variants, Mb 1679c (PE_PGRS30, 3058 amino acids) nine variants and Mb1689 (pks7, 6382 amino acids) eight variants. Except for the Mb2074c, the rate of detected mutations is not always correlated to the size of the gene.

**FIGURE 7:**
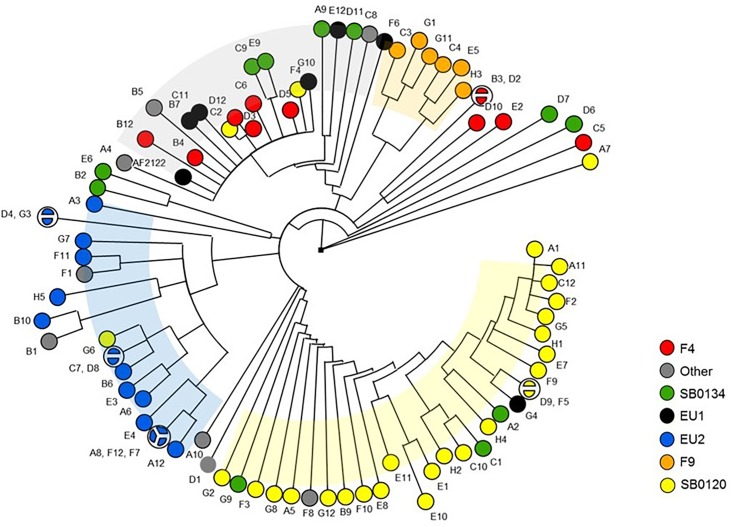
Phylogenetic tree of the *M. bovis* strains based on non-synonymous SNP of virulence genes. The Majority UPGMA tree was inferred on the 392 non-synonymous SNP detected in the 137 virulence genes sequences of the 87 French *M. bovis* isolates in comparison to the reference strain AF2122/97.

The dendrogram shown in Figure 7 highlights that nsSNPs analysis targeting virulence genes is also able to discriminate strains by group. Collectively, these results suggest that in addition to their phylogenetic differences, each group may have differential phenotypic traits.

## Discussion

In our previous study, we observed that the evolution of *M. bovis* strains responsible for tuberculosis outbreaks in France was accompanied by a significant reduction in their genetic diversity (Hauer et al., 2015). Indeed, current bTB cases in both livestock and wildlife are due to the spread of three predominant local genotypes. These observations were established by using the classical genotyping methods of spoligotyping and MLVA based on MIRU-VNTR (Hauer et al., 2016). While these targets and tools are usually sufficient for molecular epidemiology studies, they represent small hypervariable regions within the genome that generally evolve at a higher rate than the rest of the genome. Thus, in this study, we investigated the use of SNPs to define the genetic diversity extent in *M. bovis* that may provide insights into the evolution, transmission, molecular epidemiology and pathogenicity of the *M. bovis* isolates previously defined.

We selected 87 field isolates of *M. bovis* from pre-established clonal complexes. In order to identify informative SNPs among these genomes, whole genomes were sequenced and compared, while mutations were exhaustively searched.

Although these strains are genetically very close, more than 9,000 SNPs have been identified by genome comparison with commonly used filters. In some studies, SNPs were identified from a selection of genes of known functions (Behr et al., 2000). In the work of Garcia Pelayo et al. (2009), a set of 782 SNPs were identified among virulent strains of *M. bovis* and attenuated vaccine *M. bovis* BCG strains in order to better understand the genetic basis of virulence vs. attenuation. In addition, the identified SNPs have refined the phylogeny of British and French *M. bovis* strains. This set of SNPs was retested in the study by Joshi et al. (2012) to resolve the phylogenetic relationships of a panel of *M. bovis* and *M. tuberculosis* strains. Among the 300 SNP found in common with the study of Garcia Pelayo et al. (2009), 53 nsSNP were detected in a virulence gene including Mb1689 and Mb2074c most frequently mutated in this study.

In the current study, SNP-based phylogenetic analysis using the whole set of SNP is congruent with the clusters defined by spoligotyping and MLVA.

Even if phylogeny results of genotyping (combination of spoligotyping and MLVA) and SNP based analyses are concordant, some differences may exist. For instance, strains belonging to the F9-family are closely related to the SB0120 group while conventional spologtyping-MLVA linked them to the Eu1 complex. Strains previously described as part of the F4-family by spoligotype and MLVA were confirmed by SNPs analyses. A previously defined complex, European 2, is characterized by a special mutation that appears mostly in the same SNPs group.

However, to test the performance and the degree of resolution of these SNP-based typing, the panel of strains must be adapted. In this study, strains were chosen from our collection from 1983 to 2011 to represent as closely as possible the genetic diversity of French *M. bovis* strains. With other different set of strains, SNP-based genotyping should be able to resolve misclassification of clonal clusters, to identify patterns of host or spatial association, and to specify their phylogeny and genealogy.

Our results strongly suggest that many new SNP targets can be identified by comparing the genomes of field *M. bovis* strains and may be used for the development of high-resolution genotyping methods as well as other subjects in relationship to strain evolution and their transmission. This set of about 9,000 SNPs has to be reduced and detailed for a wider use.

Non-synonymous SNPs targeting virulence genes were studied to reveal potential specific phenotypic traits within well-separated phylogenetically French *M. bovis* isolates. Our analysis made it possible to identify non-synonymous SNPs in virulence genes unique to certain groups.

Interestingly, we observed that six virulence genes have between 8 and 49 SNP variations. Four of these genes (Mb1679c PE-PGRS30, Mb1849c PE_PGRS33, Mb2418 PE_PGRS34, and Mb3043c PPE46) belong to the pe/ppe family, characterized by conserved N-terminal proline-glutamate (PE) and proline-proline-glutamate (PPE) motifs, which clearly plays important roles in virulence (Fishbein et al., 2015). PE_PGRS30 has been shown to inhibit the phagolysosomal fusion (Iantomasi et al., 2012). PE_PGRS33 inhibits part of phagosome maturation of the macrophage (Dheenadhayalan et al., 2006).

The two other genes presenting most mutations (Mb1689 *pks*7 and Mb2074c *pks*12) encode polyketide synthase. These modular enzymes are required for the production of unique lipids or glycolipid conjugates, which are critical for virulence, and also for other specific components of the complex mycobacterial cell envelope. It has been reported that *pks*7 plays an important role in virulence during the persistent phase of infection (Rousseau et al., 2003). *Pks*12 was shown to encode two sets of domains needed to produce fatty acids involved in phthiocerol synthesis, the diol required for dimycocerosyl phthiocerol (DIM) synthesis (Rousseau et al., 2003; Matsunaga and Sugita, 2012).

The non-synonymous SNP detected in virulence genes are most likely to impact on protein function and contribute to phenotypic variation. Further investigations on secreted proteins, epitope recognition, or pattern of outer membrane lipids (Yruela et al., 2016; Jankute et al., 2017; Ates et al., 2018) are needed to address the consequence of these mutations in order to define phenotypic differences across the main *M. bovis* groups still responsible for the persistence of outbreaks in cattle and wild life.

Sequential chromosomal nucleotide substitutions are the main driver of *M. bovis* genome evolution if we consider that the horizontal gene transfer seems not playing a role for the evolution of contemporary modern strain (Smith, 2012; Allen et al., 2013). Likewise, the genetic changes events that can be attributed to the transposable insertion sequences such as the IS6110 was not shown to affect the core genome of *M. bovis* (Otal et al., 2008; Allen, 2017).

Differences in genome sequence, including gene families that are important for bacterial infection and transmission, highlight differences with putative functional implication between isolates otherwise classified within the same spoligotype group.

A means to refine the clonal complex identity of the different groups identified in this study could include the analysis on the strains’ pangenomes to detect the presence or absence of large deletions or insertions. To do this, it is necessary to create new reference genome sequences obtained by *de novo* assemblies of combination of second and third generation sequencing of strains belonging to main clonal groups. This would enable us to perform studies which are not limited to the core genome and to improve our knowledge on specific phenotype traits of *M. bovis* strain, the dynamics of strain transmission and evolution in multi-host systems.

## Author Contributions

All authors listed have made a substantial, direct and intellectual contribution to the work, and approved it for publication.

## Conflict of Interest Statement

The authors declare that the research was conducted in the absence of any commercial or financial relationships that could be construed as a potential conflict of interest.

## Footnotes

1 www.mbovis.org

2 http://www.megasoftware.net/

3 http://paup.phylosolutions.com/

## Supplementary Material

FIGURE S1 Geographical distribution of the 87 sequenced field isolates of *Mycobacterium bovis*. The strains were selected on the basis of their spoligotype and MLVA profile that, in different colors, represent all the genetic diversity of French *M. bovis* strains. This panel includes strains isolated from cattle (circles), goats (triangles), sheep (stars) and wildlife (losanges), pigs (pentagons), wild boar (squares).

FIGURE S2 Comparison of the homoplasy index among the different genotyping methods. HI was calculated with software package PAUP based on the number of observed changes at each character compared to the expected number of changes assuming absence of homoplasy.

TABLE S1 Metadata strain information spoligotype MLVA GenBank Number.

TABLE S2 Sequencing metrics.

TABLE S3 Genome position all SNP.

TABLE S4 Virulence genes information.

TABLE S5 Single nucleotide polymorphism on virulence gene Metadata.

TABLE S6 Virulence gene without SNP.

TABLE S7 The six virulence genes most frequently mutated.

TABLE S8 Single nucleotide polymorphism comparison to Garcia Pelayo et al. (2009) study.

## References

[B1] Allen A. R. (2017). One bacillus to rule them all? - Investigating broad range host adaptation in *Mycobacterium bovis*. *Infect. Genet. Evol.* 53 68–76. 10.1016/j.meegid.2017.04.018 28434972

[B2] Allen A. R. Dale J. McCormick C. Mallon T. R. Costello E. Gordon S. V. (2013). The phylogeny and population structure of *Mycobacterium bovis* in the British Isles. *Infect. Genet. Evol.* 20 8–15. 10.1016/j.meegid.2013.08.003 23933404

[B3] Allix C. Walravens K. Saegerman C. Godfroid J. Supply P. Fauville-Dufaux M. (2006). Evaluation of the epidemiological relevance of variable-number tandem-repeat genotyping of *Mycobacterium bovis* and comparison of the method with IS6110 restriction fragment length polymorphism analysis and spoligotyping. *J. Clin. Microbiol.* 44 1951–1962. 10.1128/JCM.01775-05 16757584 PMC1489394

[B4] Amanfu W. (2006). The situation of tuberculosis and tuberculosis control in animals of economic interest. *Tuberculosis* 86 330–335. 10.1016/j.tube.2006.01.007 16644282

[B5] Anonymous (2000). Commission decision C (2000)-4069 of 11 july 2000 amending the decision 99/467/CE establishing the officially tuberculosis-free status of bovine herds of certain member states or regions of member states. *Eur. Commun. Off. J.* L176:51.

[B6] Aranaz A. Liebana E. Mateos A. Dominguez L. Vidal D. Domingo M. (1996). Spacer oligonucleotide typing of *Mycobacterium bovis* strains from cattle and other animals: a tool for studying epidemiology of tuberculosis. *J. Clin. Microbiol.* 34 2734–2740. 8897175 10.1128/jcm.34.11.2734-2740.1996 PMC229396

[B7] Ates L. S. Dippenaar A. Sayes F. Pawlik A. Bouchier C. Ma L. (2018). Unexpected genomic and phenotypic diversity of *Mycobacterium africanum* lineage 5 affects drug resistance, protein secretion, and immunogenicity. *Genome Biol. Evol.* 10 1858–1874. 10.1093/gbe/evy145 30010947 PMC6071665

[B8] Behr M. A. Schroeder B. G. Brinkman J. N. Slayden R. A. Barry CE 3rd (2000). A point mutation in the mma3 gene is responsible for impaired methoxymycolic acid production in *Mycobacterium bovis* BCG strains obtained after 1927. *J. Bacteriol.* 182 3394–3399. 10852869 10.1128/jb.182.12.3394-3399.2000 PMC101902

[B9] Biek R. O’Hare A. Wright D. Mallon T. McCormick C. Orton R. J. (2012). Whole genome sequencing reveals local transmission patterns of *Mycobacterium bovis* in sympatric cattle and badger populations. *PLoS Pathog.* 8:e1003008. 10.1371/journal.ppat.1003008 23209404 PMC3510252

[B10] Blouin Y. Hauck Y. Soler C. Fabre M. Vong R. Dehan C. (2012). Significance of the identification in the horn of Africa of an exceptionally deep branching *Mycobacterium tuberculosis* clade. *PLoS One* 7:e52841. 10.1371/journal.pone.0052841 23300794 PMC3531362

[B11] Bolger A. M. Lohse M. Usadel B. (2014). Trimmomatic: a flexible trimmer for Illumina sequence data. *Bioinformatics* 30 2114–2120. 10.1093/bioinformatics/btu170 24695404 PMC4103590

[B12] Boniotti M. B. Goria M. Loda D. Garrone A. Benedetto A. Mondo A. (2009). Molecular typing of *Mycobacterium bovis* strains isolated in Italy from 2000 to 2006 and evaluation of variable-number tandem repeats for geographically optimized genotyping. *J. Clin. Microbiol.* 47 636–644. 10.1128/JCM.01192-08 19144792 PMC2650904

[B13] Corner L. A. Trajstman A. C. Lund K. (1995). Determination of the optimum concentration of decontaminants for the primary isolation of *Mycobacterium bovis*. *N. Z. Vet. J.* 43 129–133. 10.1080/00480169.1995.35871 16031831

[B14] Dheenadhayalan V. Delogu G. Brennan M. J. (2006). Expression of the PE_PGRS 33 protein in Mycobacterium smegmatis triggers necrosis in macrophages and enhanced mycobacterial survival. *Microbes Infect.* 8 262–272. 10.1016/j.micinf.2005.06.021 16203168

[B15] Fishbein S. van Wyk N. Warren R. M. Sampson S. L. (2015). Phylogeny to function: PE/PPE protein evolution and impact on *Mycobacterium tuberculosis* pathogenicity. *Mol. Microbiol.* 96 901–916. 10.1111/mmi.12981 25727695

[B16] Forrellad M. A. Klepp L. I. Gioffre A. Sabio Y. Garcia J. Morbidoni H. R. (2013). Virulence factors of the *Mycobacterium tuberculosis* complex. *Virulence* 4 3–66. 10.4161/viru.22329 23076359 PMC3544749

[B17] Frothingham R. Meeker-O’Connell W. A. (1998). Genetic diversity in the *Mycobacterium tuberculosis* complex based on variable numbers of tandem DNA repeats. *Microbiology* 144(Pt 5), 1189–1196. 9611793 10.1099/00221287-144-5-1189

[B18] Garcia Pelayo M. C. Uplekar S. Keniry A. Mendoza Lopez P. Garnier T. Nunez Garcia J. (2009). A comprehensive survey of single nucleotide polymorphisms (SNPs) across *Mycobacterium bovis* strains and *M. bovis* BCG vaccine strains refines the genealogy and defines a minimal set of SNPs that separate virulent *M. bovis* strains and *M. bovis* BCG strains. *Infect. Immun.* 77 2230–2238. 10.1128/IAI.01099-08 19289514 PMC2681724

[B19] Gardy J. L. Johnston J. C. Ho Sui S. J. Cook V. J. Shah L. Brodkin E. (2011). Whole-genome sequencing and social-network analysis of a tuberculosis outbreak. *N. Engl. J. Med.* 364 730–739. 10.1056/NEJMoa1003176 21345102

[B20] Haddad N. Ostyn A. Karoui C. Masselot M. Thorel M. F. Hughes S. L. (2001). Spoligotype diversity of *Mycobacterium bovis* strains isolated in France from 1979 to 2000. *J. Clin. Microbiol.* 39 3623–3632. 10.1128/JCM.39.10.3623-3632.2001 11574583 PMC88399

[B21] Hauer A. De Cruz K. Cochard T. Godreuil S. Karoui C. Henault S. (2015). Genetic evolution of *Mycobacterium Bovis* causing tuberculosis in Livestock and wildlife in France since 1978. *PLoS One* 10:e0117103. 10.1371/journal.pone.0117103 25658691 PMC4319773

[B22] Hauer A. Michelet L. De Cruz K. Cochard T. Branger M. Karoui C. (2016). MIRU-VNTR allelic variability depends on *Mycobacterium bovis* clonal group identity. *Infect. Genet. Evol.* 45 165–169. 10.1016/j.meegid.2016.08.038 27594144

[B23] Homolka S. Projahn M. Feuerriegel S. Ubben T. Diel R. Nubel U. (2012). High resolution discrimination of clinical *Mycobacterium tuberculosis* complex strains based on single nucleotide polymorphisms. *PLoS One* 7:e39855. 10.1371/journal.pone.0039855 22768315 PMC3388094

[B24] Huard R. C. Fabre M. de Haas P. Lazzarini L. C. van Soolingen D. Cousins D. (2006). Novel genetic polymorphisms that further delineate the phylogeny of the *Mycobacterium tuberculosis* complex. *J. Bacteriol.* 188 4271–4287. 10.1128/JB.01783-05 16740934 PMC1482959

[B25] Iantomasi R. Sali M. Cascioferro A. Palucci I. Zumbo A. Soldini S. (2012). PE_PGRS30 is required for the full virulence of *Mycobacterium tuberculosis*. *Cell Microbiol.* 14 356–367. 10.1111/j.1462-5822.2011.01721.x 22050772

[B26] Imai T. Ohta K. Kigawa H. Kanoh H. Taniguchi T. Tobari J. (1994). Preparation of high-molecular-weight DNA: application to mycobacterial cells. *Anal. Biochem.* 222 479–482. 10.1006/abio.1994.1520 7864376

[B27] Jankute M. Nataraj V. Lee O. Y. Wu H. H. T. Ridell M. Garton N. J. (2017). The role of hydrophobicity in tuberculosis evolution and pathogenicity. *Sci. Rep.* 7:1315. 10.1038/s41598-017-01501-0 28465507 PMC5431016

[B28] Joshi D. Harris N. B. Waters R. Thacker T. Mathema B. Krieswirth B. (2012). Single nucleotide polymorphisms in the *Mycobacterium bovis* genome resolve phylogenetic relationships. *J. Clin. Microbiol.* 50 3853–3861. 10.1128/JCM.01499-12 22993186 PMC3502966

[B29] Joshi S. M. Pandey A. K. Capite N. Fortune S. M. Rubin E. J. Sassetti C. M. (2006). Characterization of mycobacterial virulence genes through genetic interaction mapping. *Proc. Natl. Acad. Sci. U.S.A.* 103 11760–11765. 10.1073/pnas.0603179103 16868085 PMC1544243

[B30] Matsunaga I. Sugita M. (2012). Mycoketide: a CD1c-presented antigen with important implications in mycobacterial infection. *Clin. Dev. Immunol.* 2012:981821. 10.1155/2012/981821 22536277 PMC3318773

[B31] Muller B. Durr S. Alonso S. Hattendorf J. Laisse C. J. Parsons S. D. (2013). Zoonotic *Mycobacterium bovis*-induced tuberculosis in humans. *Emerg. Infect. Dis.* 19 899–908. 10.3201/eid1906.120543 23735540 PMC4816377

[B32] Muller B. Hilty M. Berg S. Garcia-Pelayo M. C. Dale J. Boschiroli M. L. (2009). African 1, an epidemiologically important clonal complex of *Mycobacterium bovis* dominant in Mali, Nigeria, Cameroon, and Chad. *J. Bacteriol.* 191 1951–1960. 10.1128/JB.01590-08 19136597 PMC2648362

[B33] Otal I. Gomez A. B. Kremer K. de Haas P. Garcia M. J. Martin C. (2008). Mapping of IS6110 insertion sites in *Mycobacterium bovis* isolates in relation to adaptation from the animal to human host. *Vet. Microbiol.* 129 333–341. 10.1016/j.vetmic.2007.11.038 18207337

[B34] Palmer M. V. Welsh M. D. Hostetter J. M. (2012). Mycobacterial diseases of animals 2012. *Vet. Med. Int.* 2012:684720. 10.1155/2012/684720 23213628 PMC3505661

[B35] Rodriguez S. Romero B. Bezos J. de Juan L. Alvarez J. Castellanos E. (2010). High spoligotype diversity within a *Mycobacterium bovis* population: clues to understanding the demography of the pathogen in Europe. *Vet. Microbiol.* 141 89–95. 10.1016/j.vetmic.2009.08.007 19720476

[B36] Rodriguez-Campos S. Aranaz A. de Juan L. Saez-Llorente J. L. Romero B. Bezos J. (2011). Limitations of spoligotyping and variable-number tandem-repeat typing for molecular tracing of *Mycobacterium bovis* in a high-diversity setting. *J. Clin. Microbiol.* 49 3361–3364. 10.1128/JCM.00301-11 21752973 PMC3165628

[B37] Rodriguez-Campos S. Navarro Y. Romero B. de Juan L. Bezos J. Mateos A. (2013). Splitting of a prevalent *Mycobacterium bovis* spoligotype by variable-number tandem-repeat typing reveals high heterogeneity in an evolving clonal group. *J. Clin. Microbiol.* 51 3658–3665. 10.1128/JCM.01271-13 23985914 PMC3889748

[B38] Rodriguez-Campos S. Schurch A. C. Dale J. Lohan A. J. Cunha M. V. Botelho A. (2012). European 2–a clonal complex of *Mycobacterium bovis* dominant in the Iberian Peninsula. *Infect. Genet. Evol.* 12 866–872. 10.1016/j.meegid.2011.09.004 21945286

[B39] Rodriguez-Campos S. Smith N. H. Boniotti M. B. Aranaz A. (2014). Overview and phylogeny of *Mycobacterium tuberculosis* complex organisms: implications for diagnostics and legislation of bovine tuberculosis. *Res. Vet. Sci.* 97(Suppl.), S5–S19. 10.1016/j.rvsc.2014.02.009 24630673

[B40] Roetzer A. Diel R. Kohl T. A. Ruckert C. Nubel U. Blom J. (2013). Whole genome sequencing versus traditional genotyping for investigation of a *Mycobacterium tuberculosis* outbreak: a longitudinal molecular epidemiological study. *PLoS Med.* 10:e1001387. 10.1371/journal.pmed.1001387 23424287 PMC3570532

[B41] Roring S. Scott A. Brittain D. Walker I. Hewinson G. Neill S. (2002). Development of variable-number tandem repeat typing of *Mycobacterium bovis*: comparison of results with those obtained by using existing exact tandem repeats and spoligotyping. *J. Clin. Microbiol.* 40 2126–2133. 12037076 10.1128/JCM.40.6.2126-2133.2002 PMC130792

[B42] Rousseau C. Sirakova T. D. Dubey V. S. Bordat Y. Kolattukudy P. E. Gicquel B. (2003). Virulence attenuation of two Mas-like polyketide synthase mutants of *Mycobacterium tuberculosis*. *Microbiology* 149(Pt 7), 1837–1847. 10.1099/mic.0.26278-0 12855735

[B43] Sassetti C. M. Rubin E. J. (2003). Genetic requirements for mycobacterial survival during infection. *Proc. Natl. Acad. Sci. U.S.A.* 100 12989–12994. 10.1073/pnas.2134250100 14569030 PMC240732

[B44] Skuce R. A. McDowell S. W. Mallon T. R. Luke B. Breadon E. L. Lagan P. L. (2005). Discrimination of isolates of *Mycobacterium bovis* in Northern Ireland on the basis of variable numbers of tandem repeats (VNTRs). *Vet. Rec.* 157 501–504. 16244231 10.1136/vr.157.17.501

[B45] Smith N. H. (2012). The global distribution and phylogeography of *Mycobacterium bovis* clonal complexes. *Infect. Genet. Evol.* 12 857–865. 10.1016/j.meegid.2011.09.007 21945588

[B46] Smith N. H. Gordon S. V. de la Rua-Domenech R. Clifton-Hadley R. S. Hewinson R. G. (2006a). Bottlenecks and broomsticks: the molecular evolution of *Mycobacterium bovis*. *Nat. Rev. Microbiol.* 4 670–681. 10.1038/nrmicro1472 16912712

[B47] Smith N. H. Kremer K. Inwald J. Dale J. Driscoll J. R. Gordon S. V. (2006b). Ecotypes of the *Mycobacterium tuberculosis* complex. *J. Theor. Biol.* 239 220–225. 10.1016/j.jtbi.2005.08.036 16242724

[B48] Smith N. H. Hewinson R. G. Kremer K. Brosch R. Gordon S. V. (2009). Myths and misconceptions: the origin and evolution of *Mycobacterium tuberculosis*. *Nat. Rev. Microbiol.* 7 537–544. 10.1038/nrmicro2165 19483712

[B49] Supply P. Allix C. Lesjean S. Cardoso-Oelemann M. Rusch-Gerdes S. Willery E. (2006). Proposal for standardization of optimized mycobacterial interspersed repetitive unit-variable-number tandem repeat typing of *Mycobacterium tuberculosis*. *J. Clin. Microbiol.* 44 4498–4510. 10.1128/JCM.01392-06 17005759 PMC1698431

[B50] Tamura K. Dudley J. Nei M. Kumar S. (2007). MEGA4: molecular evolutionary genetics analysis (MEGA) software version 4.0. *Mol. Biol. Evol.* 24 1596–1599. 10.1093/molbev/msm092 17488738

[B51] Wilgenbusch D. (2003). Inferring evolutionary trees with PAUP^∗^. *Curr. Protoc. Bioinform.* 6.4.1–6.4.28. 10.1002/0471250953.bi0604s00 18428704

[B52] Yruela I. Contreras-Moreira B. Magalhaes C. Osorio N. S. Gonzalo-Asensio J. (2016). *Mycobacterium tuberculosis* complex exhibits lineage-specific variations affecting protein ductility and epitope recognition. *Genome Biol. Evol.* 8 3751–3764. 10.1093/gbe/evw279 28062754 PMC5521731

[B53] Zhang J. Abadia E. Refregier G. Tafaj S. Boschiroli M. L. Guillard B. (2010). *Mycobacterium tuberculosis* complex CRISPR genotyping: improving efficiency, throughput and discriminative power of ’spoligotyping’ with new spacers and a microbead-based hybridization assay. *J. Med. Microbiol.* 59(Pt 3), 285–294. 10.1099/jmm.0.016949-0 19959631

