# Supplementary figures and images for: Accurate Phylogenetic Relationships Among Mycobacterium bovis Strains Circulating in France Based on Whole Genome Sequencing and Single Nucleotide Polymorphism Analysis
Source: Front Microbiol. 2019 May 3;10:955. doi: 10.3389/fmicb.2019.00955 (PMC6509552; doi:10.3389/fmicb.2019.00955)

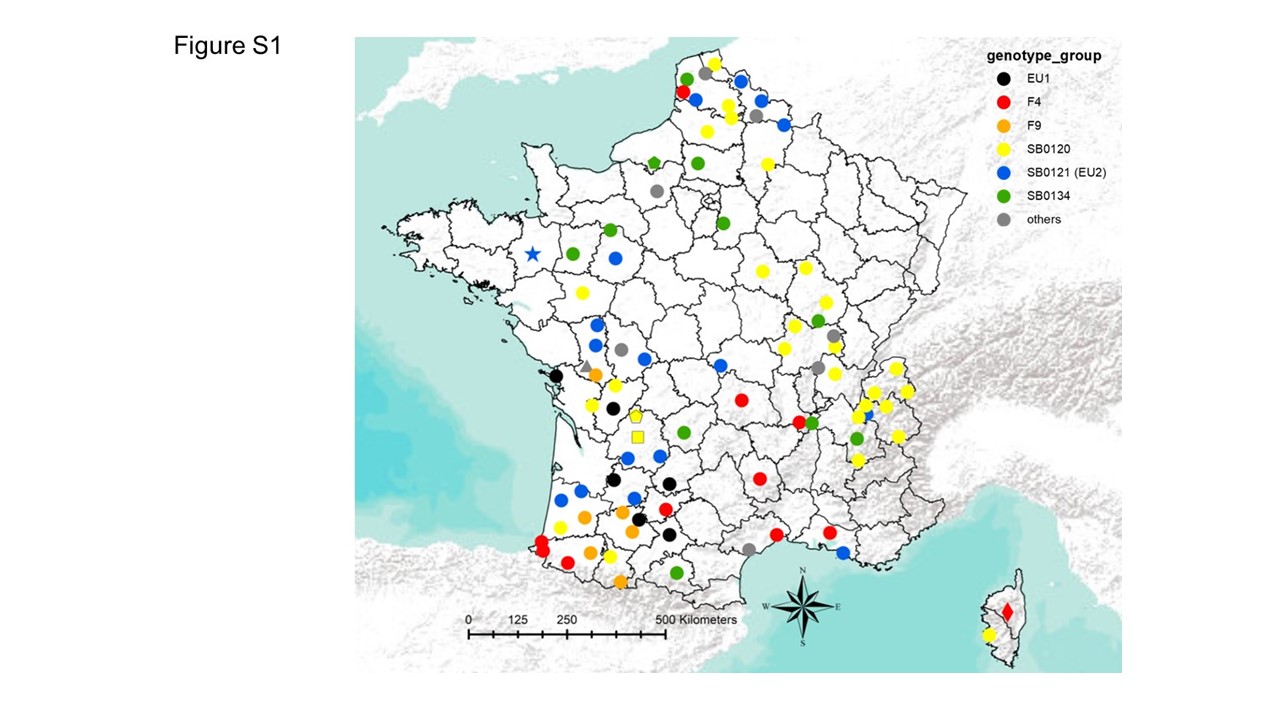

Supplement: FIGURE S1 — Geographical distribution of the 87 sequenced field isolates of Mycobacterium bovis. The strains were selected on the basis of their spoligotype and MLVA profile that, in different colors, represent all the genetic diversity of French M. bovis strains. This panel includes strains isolated from cattle (circles), goats (triangles), sheep (stars) and wildlife (losanges), pigs (pentagons), wild boar (squares). [file Image_1.JPEG]

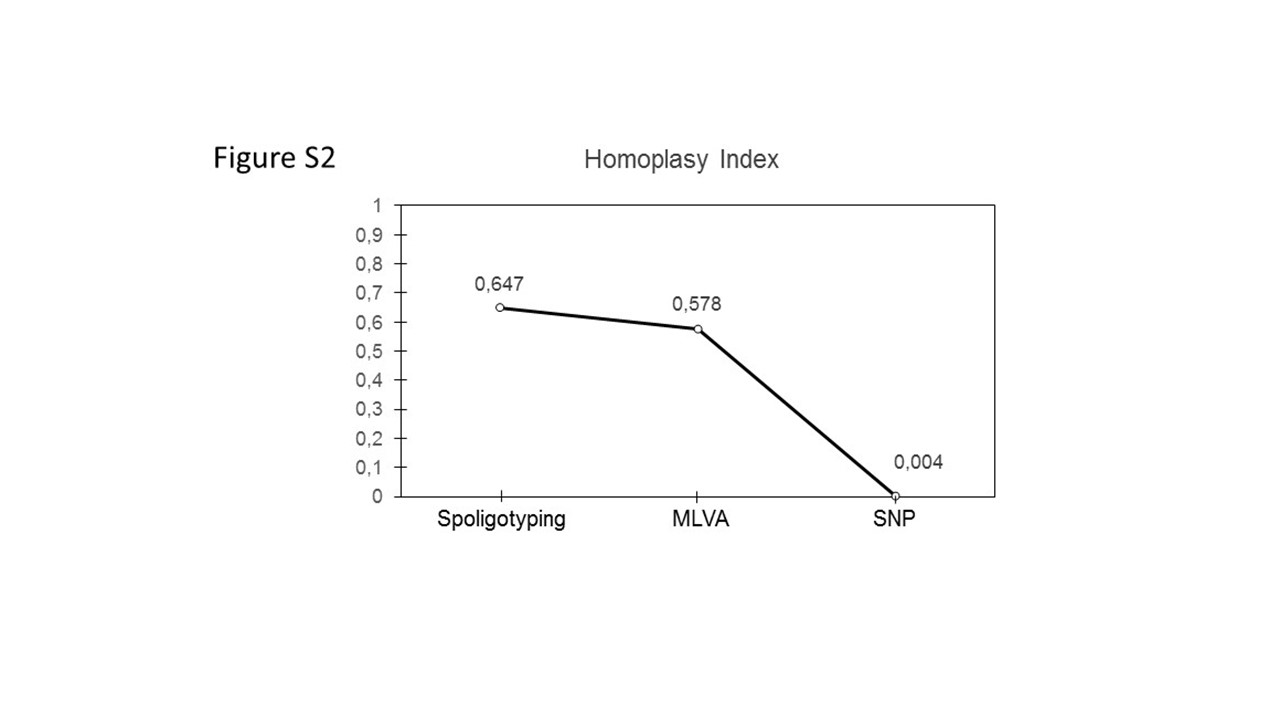

Supplement: FIGURE S2 — Comparison of the homoplasy index among the different genotyping methods. HI was calculated with software package PAUP based on the number of observed changes at each character compared to the expected number of changes assuming absence of homoplasy. [file Image_2.JPEG]
